# Activation of the G_i_ protein-RHOA axis by non-canonical Hedgehog signaling is independent of primary cilia

**DOI:** 10.1371/journal.pone.0203170

**Published:** 2018-08-27

**Authors:** Lan Ho Wei, Mohammad Arastoo, Ioanna Georgiou, David R. Manning, Natalia A. Riobo-Del Galdo

**Affiliations:** 1 Department of Biochemistry and Molecular Biology, Thomas Jefferson University, Philadelphia, Pennsylvania, United States of America; 2 Leeds Institute of Cancer and Pathology, University of Leeds, Leeds, United Kingdom; 3 Department of Systems Pharmacology and Translational Therapeutics, University of Pennsylvania Perelman School of Medicine, Philadelphia, Pennsylvania, United States of America; 4 School of Molecular and Cellular Biology, University of Leeds, Leeds, United Kingdom; National Cancer Center, JAPAN

## Abstract

Primary cilia are solitary organelles that emanate from the plasma membrane during growth arrest in almost all mammalian cells. The canonical Hedgehog (HH) pathway requires trafficking of the G protein-coupled receptor SMOOTHENED (SMO) and the GLI transcription factors to the primary cilium upon binding of a HH ligand to PATCHED1. However, it is unknown if activation of the small GTPase RHOA by SMO coupling to heterotrimeric G_i_ proteins, a form of non-canonical HH signaling, requires localization of SMO in the primary cilium. In this study, we compared RHOA and G_i_ protein stimulation by activation of SMO or sphingosine 1-phosphate receptor (S1P) receptors in WT and KIF3A-deficient mouse embryonic fibroblasts that lack primary cilia. We found that activation of SMO in response to Sonic HH (SHH) or purmorphamine (PUR), a small molecule agonist of SMO, stimulates G_i_ proteins and RHOA independently of the presence of primary cilia, similar to the effects of S1P. However, while S1P induced a fast activation of AKT that is sensitive to the G_i_ inhibitor pertussis toxin, HH pathway activators did not significantly activate AKT, suggesting that RHOA activation is not downstream of AKT. Our findings demonstrate that early events in some forms of non-canonical HH signaling occur in extraciliary membranes, which might be particularly relevant for actively-cycling cells, for some cancers characterized by loss of primary cilia, and in ciliopathies.

## Introduction

HEDGEHOG (HH) signaling is essential during embryonic development and postnatal tissue homeostasis. Its dysregulation is associated with severe developmental defects and cancer [1]. HH ligands, such as Sonic Hedgehog (SHH), stimulate the activity of the GLI2 and GLI3 transcription factors to induce expression of GLI-target genes, among which is the highly active and short-lived GLI1 isoform [2]. The HH proteins bind to a 12-transmembrane (TM) receptor called PATCHED1 (PTCH1), derepressing the 7-TM protein SMOOTHENED (SMO), which acts as the central transducer of the HH pathway. Stimulation of GLI transcriptional activity requires SMO accumulation at the primary cilium through fusion of vesicles containing SMO to the plasma membrane followed by lateral diffusion [3]. The primary cilium is an immotile flagellar-like organelle containing nine microtubule duplets and a specialized protein and phospholipid composition. Ciliogenesis occurs by tubulin polymerization in the plus end of the basal body. Thus, the presence of primary cilia is observed exclusively during interphase and in quiescent cells. Assembly, maintenance and signaling in cilia require intraflagellar transport (IFT), a bidirectional movement of cargo by the action of molecular motors. Mutations that impair anterograde or retrograde IFT impair GLI activation in response to SHH, causing a range of developmental phenotypes similar to SMO loss of function [4–6]. KIF3A is a subunit of the heterotrimeric kinesin-II motor that participates in anterograde IFT and is essential for ciliogenesis. KIF3A deficiency in mice is embryonic lethal and results in situs inversus and neural tube and limb formation defects by impairment of canonical HH signaling [5].

Our previous work established that SMO is a G protein-coupled receptor (GPCR) with selectivity towards the G_i_ family of G proteins [7, 8]. Activation of G_i_ by SMO leads to reduction in cytoplasmic cAMP concentration by inhibition of adenylate cyclases and consequent reduction of cAMP-dependent protein kinase (PKA) activity [8–10]. Since PKA is a negative regulator of the canonical HH pathway via phosphorylation of GLI2 and GLI3, which targets them for processing into transcriptional repressors [11,12], activation of G_i_ by SMO is believed to facilitate GLI activation. Indeed, coupling of SMO to G_i_ is necessary for canonical HH signaling in some albeit not all cell types, suggesting that basal cAMP levels, determined by the presence of other growth factors or hormones that regulate cAMP, may underlie the differential requirement of G_i_ [7, 13].

In addition to GLI activation, SMO also stimulates G_i_-dependent rapid signaling cascades that are sensitive to a *Bordetella pertussis* toxin (PTX) [14, 15]. Among them is the activation of the small GTPases RHOA and RAC1 in fibroblasts and endothelial cells by SMO, resulting in actin cytoskeleton reorganization and promotion of migration and tubulogenesis, respectively [13,16]. Activation of RHOA and RAC1 is sensitive to PTX and a phosphoinositide-3-kinase (PI3K) inhibitor [13,16]. These GLI-independent roles of HH proteins are collectively known as “non-canonical” type II HH signaling and occur rapidly after addition of stimuli, typically within minutes [14,15]. Interestingly, in NIH 3T3 cells the slow-acting canonical HH pathway is also dependent on G_i_ and PI3K activity, more specifically on the PI3K effector AKT [7,11], suggesting that non-canonical signaling could be an early step in the pathway leading to GLI activation. That non-canonical signaling is sufficient in this regard, however, is precluded by the observation that an oncogenic SMO mutant lacking the cilia localization sequence (SMOM2-CLD) could restore RHOA and RAC1 activation in *Smo*^-/-^ MEFs despite being unable to induce GLI-target genes [13]. The apparent dispensability of primary cilia with respect to non-canonical signaling supports the alternative hypothesis that non-canonical HH signaling involves an extraciliary pool of SMO and can occur in parallel to canonical signaling initiated by ciliary SMO. Here, we tested that hypothesis by investigating the ability of SHH and a synthetic SMO agonist to stimulate G_i_ proteins and RHOA through endogenous SMO in wild-type (WT) vs. *Kif3a*^*-/-*^ mouse embryonic fibroblasts (MEFs). Our results indicate that primary cilia are not required for RHOA activation by SMO or for reduction of cAMP levels, a consequence of G_i_ activation. We also found that while PI3K is necessary for RHOA activation, activation of AKT over basal tonic levels is not. Throughout our study, we compared the activity of SMO agonists with sphingosine 1-phosphate (S1P), which activates G_i_- and G_13_-coupled receptors in MEFs [17–20]. We found that S1P also stimulates RHOA and reduces cAMP in both WT and *Kif3a*^-/-^ MEFs; however, S1P induces acute phosphorylation of AKT in a G_i_-dependent manner unlike SHH or PUR.

Our results conclusively demonstrate that primary cilia are dispensable for non-canonical Hh signaling leading to SMO/G_i_ coupling and RHOA activation, and support the notion of that simultaneous stimulation of ciliary and extraciliary pools of SMO exert separate functions. In addition, these findings highlight the existence of similarities and differences in the early downstream signaling signature of S1P and HH pathway agonists, despite the common involvement of G_i_ proteins.

## Results

### Primary cilia are dispensable for RHOA activation by Smoothened

**Fig 1:**
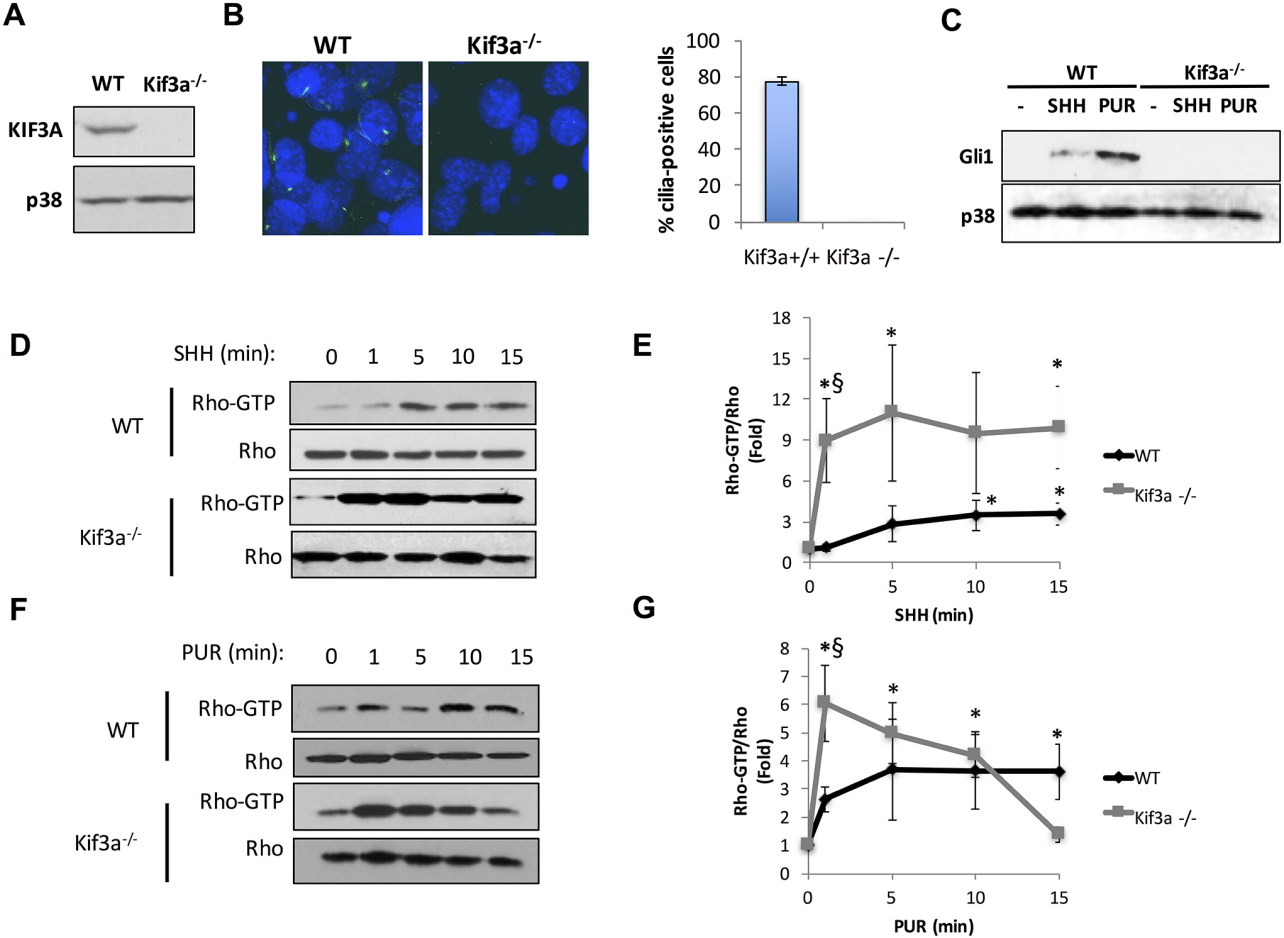
RHOA activation is a cilium-independent response to Hh pathway agonists. **A**. Representative Western blot of endogenous KIF3A expression in whole cell lysates from isogenic MEFs, *Kif3a*^+/+^ (WT) MEFs and *Kif3a*^-/-^ MEFs. p38 was used as a loading control (n = 3). **B**. Staining of primary cilia with anti-acetylated α-tubulin (green channel) after 48 h serum starvation in WT and *Kif3a*^-/-^ MEFs. Nuclei were counterstained with DAPI. The bar graph shows the mean % ± SEM of ciliated cells in both genotypes (n = 3). **C**. GLI1 induction in WT and *Kif3a*^-/-^ MEFs after 24h serum starvation followed by 48 h incubation in the presence of vehicle (-), 2.5 μg/ml SHH, or 5 μM PUR. Representative experiment of 3 independent repeats. **D**. RHOA pulldown assays in WT and *Kif3a*^-/-^ MEFs serum starved for 24 h followed by addition of 2.5 μg/ml SHH for 0–15 min. **E**. Densitometric quantification of RHOA-GTP/total RHOA increase in response to SHH at the indicated times. The ratios were expressed as fold change compared to t = 0. *p < 0.*05*; n = 4. The ratio of RHOA-GTP/total RHOA was also significantly higher in *Kif3a*^-/-^ than WT MEFs at 1 min (§ p < 0.05; Student’s *t* test). **F**. RHOA pulldown assays in WT and *Kif3a*^-/-^ MEFs serum starved for 24 h followed by addition of 5 μM PUR for 0–15 min. **G**. Densitometric quantification of RHOA-GTP/total RHOA increase in response to PUR at the indicated times. The ratios were expressed as fold change compared to t = 0. *p < 0.*05* vs. t = 0; n = 3. The ratio of RHOA-GTP/total RHOA was also significantly higher in *Kif3a*^-/-^ MEFs at 1 min (§ p < 0.05; Student’s *t* test).

**Fig 2:**
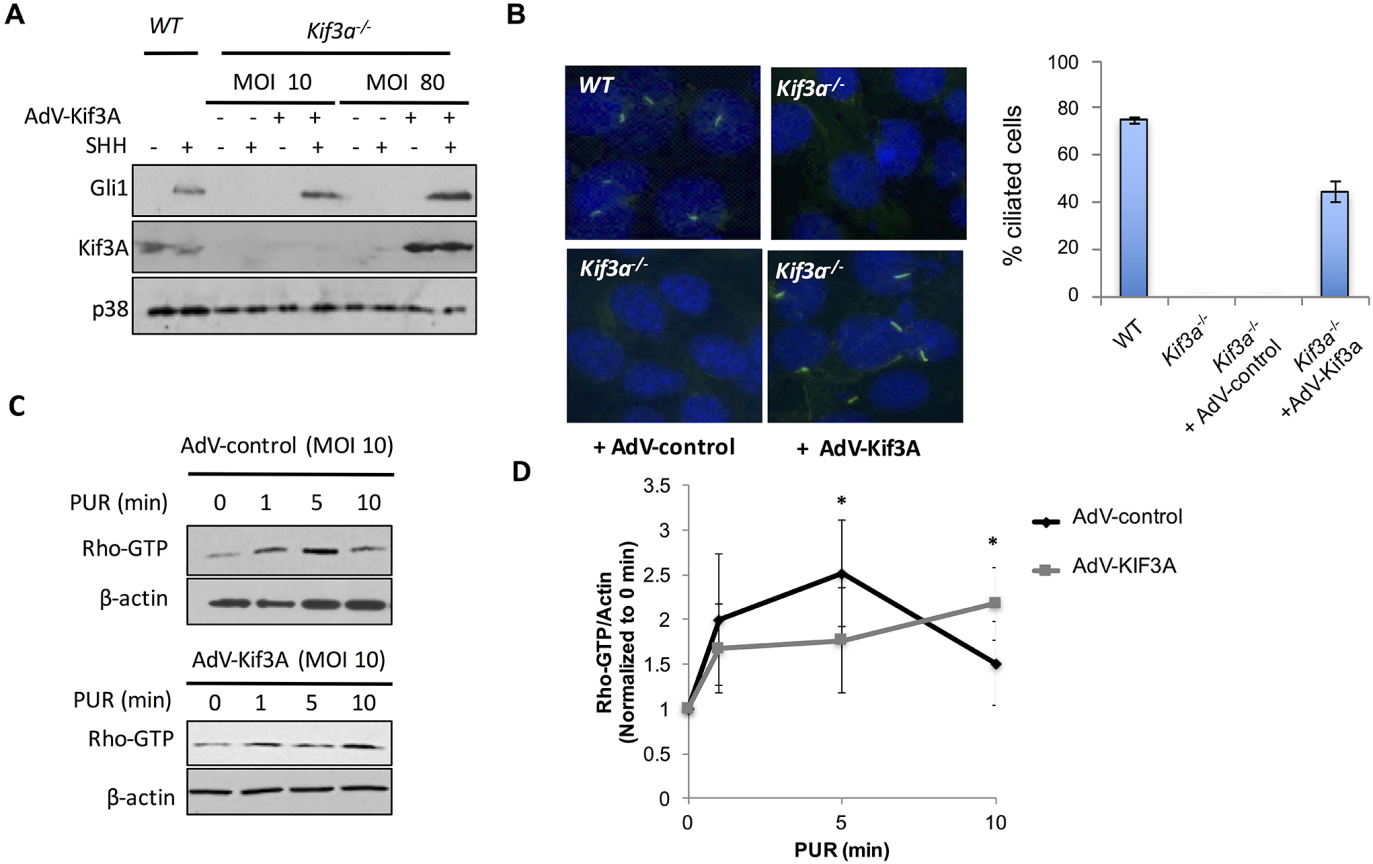
Rescue of KIF3A expression in *Kif3a*^-/-^ MEFs does not attenuate RHOA activation mediated by purmorphamine. **A**. GLI1 induction and KIF3A expression in WT MEFs (WT) vs. *Kif3a*^-/-^ MEFs infected with AdV-Kif3a at MOIs of 10 and 80. p38 was used as loading control (n = 3). **B**. Staining of primary cilia in WT MEFs, *Kif3a*^-/-^ MEFs, or *Kif3a*^-/-^ MEFs infected with either control (empty) AdV or AdV-Kif3a (MOI = 10). Cells were stained with anti-acetylated α-tubulin and anti-Alexa 488 to visualize the axoneme of each primary cilium. Quantification of the percentage of cilia-positive cells (mean ± S.E.M; n = 4) **C**. RHOA activation in *Kif3a*^-/-^ MEFs infected with control AdV or AdV-Kif3a at MOI = 10 and stimulated with 5 μM PUR for the indicated times (β-actin was used as loading control because total RHOA was obscured by a cross-reactive protein that appears as a consequence of AdV infection). **D**. Densitometric quantification of RHOA-GTP/ β-actin increase in response to 5 μM PUR at the indicated times. The ratios were expressed as fold change compared to t = 0. * *p < 0*.*05*; n = 3.

In order to determine if primary cilia are necessary for RHOA activation by non-canonical HH signaling, we compared activation of RHOA in MEFs isolated from WT or *Kif3a*^*-/-*^ mice. We first verified that *Kif3a*^*-/-*^ MEFs effectively do not express KIF3A (Fig 1A) and fail to form primary cilia upon serum starvation, unlike WT MEFs, as determined by immunofluorescence staining of the ciliary axoneme using an antibody against acetylated α-tubulin (Fig 1B). Neither SHH nor the SMO small molecule agonist purmorphamine (PUR) induced GLI1 expression in *Kif3a*^*-/-*^ MEFs, while GLI1 expression was observed in WT cells (Fig 1C). Despite the inability of endogenous SMO to support activation of the canonical HH pathway in *Kif3a*^*-/-*^ MEFs, both SHH and PUR activated RHOA in the absence of cilia (Fig 1D and 1G). Moreover, the magnitude of RHOA activation at 1 min was larger in *Kif3a*^*-/-*^ MEFs than in WT MEFs. Previous reports indicated that *Kif3a*^*-/-*^ MEFs had a faster migratory response to SHH [21]. The greater degree of activation at 1 min and the faster migratory responses suggest either that the primary cilium exerts a negative regulation on small GTPase stimulation by SMO, perhaps by competition between a ciliary and extraciliary pool of SMO, or that clonal differences exist between the two types of MEFs. If the former were the case, restoration of primary cilia formation in *Kif3a*^-/-^ MEFs should decrease the magnitude of RHOA activation. We therefore introduced myc-tagged KIF3A in *Kif3a*^*-/-*^ MEFs by adenoviral (AdV) delivery. The AdV-Kif3a rescued KIF3A expression, restored the capacity to form primary cilia upon serum starvation, and restored GLI1 induction in response to SHH, while a control AdV did not (Fig 2A and 2B). Notably, AdV-Kif3a restored primary cilia and canonical HH signaling at a multiplicity of infection (MOI = 10) that resulted in KIF3A expression below endogenous KIF3A levels in WT MEFs (Fig 2A and 2B). *Kif3a*^*-/-*^ MEFs transduced with AdV-Kif3a or AdV-control at MOIs of 10 showed no consistent difference in the capacity of PUR to stimulate RHOA (Fig 2C and 2D), suggesting that the presence of primary cilia does not diminish non-canonical HH signaling.

Next, we assessed the role of primary cilia on the ability of a different G_i_-coupled receptor to activate RHOA. The S1P receptors S1PR1, S1PR2, and S1PR3, expressed endogenously in MEFs, couple to both G_i_ and G_13_/G_q_ proteins in MEFs [17,19]. Stimulation of WT MEFs with S1P resulted in a significant increase in RHOA-GTP levels (S1 Fig), consistent with published data [18–19]. S1P similarly activated RHOA in *Kif3a*^*-/-*^ MEFs, indicating that primary cilia are dispensable for this event (S1 Fig).

### Smoothened can couple to G_i_ proteins outside primary cilia

**Fig 3:**
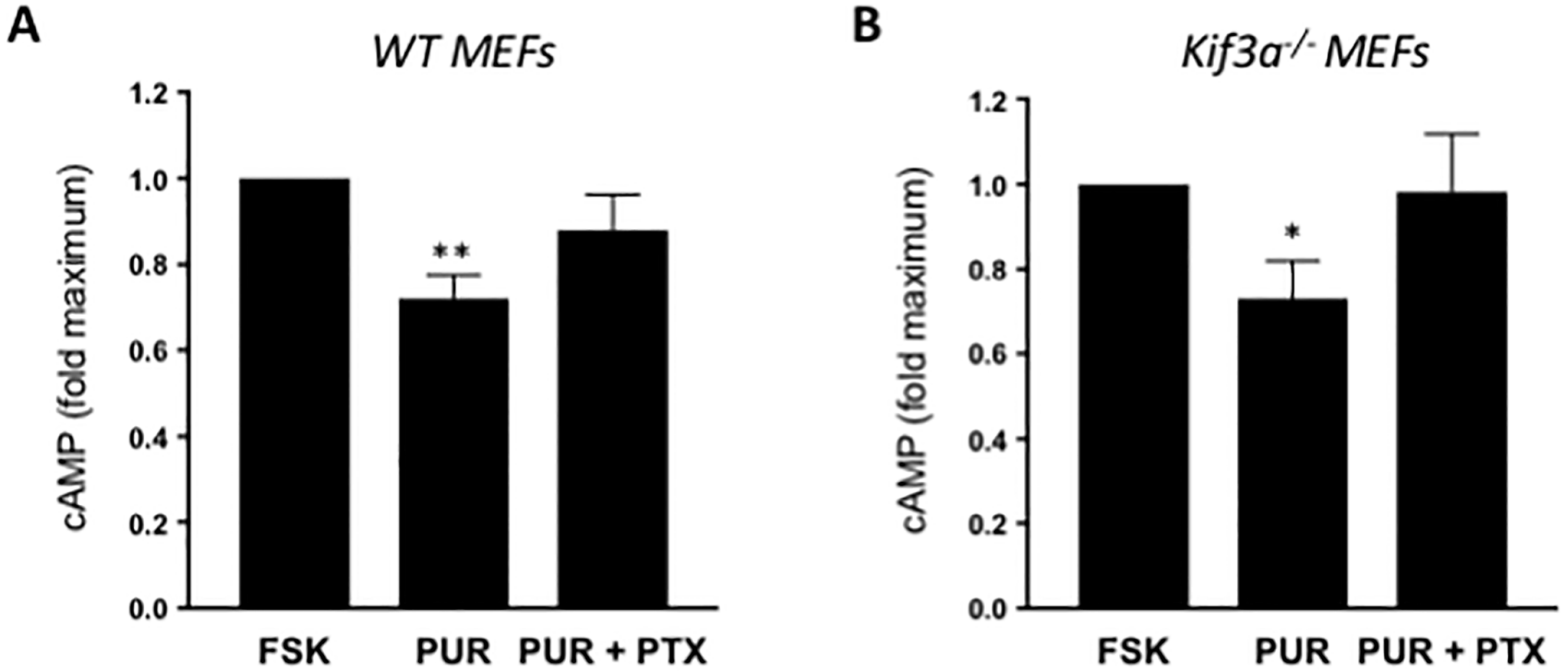
Purmorphamine decreases FSK-directed cAMP production in a cilium-independent manner. **(A)** WT MEFs and **(B)** Kif3a^-/-^ MEFs were serum-starved at sub-confluency for 24 h in the presence or absence of 200 ng/ml PTX before treatment with 20 μM forskolin (FSK) or a combination of FSK and 5 μM PUR for 10 min. Lysates were subjected to cAMP EIA as described by the manufacturer. Bars represent mean ± S.E.M. of FSK-induced cAMP production *p < 0.05; **p<0.*01* (n = 4).

Since activation of RHOA by SMO does not require the primary cilium and we previously demonstrated that it is mediated by heterotrimeric G_i_ proteins [13], we reasoned that coupling of SMO to G_i_ is also independent of primary cilia. To test this hypothesis, we measured the capacity of PUR to reduce cAMP levels in WT vs. *Kif3a*^*-/-*^ MEFs. Because basal cAMP levels are too low in MEFs to detect a reduction after G_i_ activation, we first stimulated the production of cAMP with forskolin (FSK), an adenylyl cyclase activator. PUR decreased the maximal cAMP production in response to FSK in both WT and *Kif3a*^*-/-*^ MEFs, but not in cells pre-treated with the G_i_ inhibitor PTX (Fig 3A and 3B). These results suggest that SMO reduces cAMP outside the primary cilium, through stimulation of a PTX-sensitive heterotrimeric G_i_ protein. Co-stimulation of WT MEFs with S1P and FSK resulted in a comparable reduction in cAMP production that was also prevented by PTX (S2 Fig). Even though S1P receptors can couple to other G proteins besides G_i_, the inhibition of adenylyl cyclase activity is a reasonably exclusive read-out of G_i_ activation. Altogether, our data demonstrate that SMO can efficiently couple to G_i_ proteins in extracilliary membranes and that the magnitude of this effect is comparable to another G_i_-coupled GPCR.

### Divergence of AKT activation profiles between Smoothened and sphingosine-1-phosphate receptors

**Fig 4:**
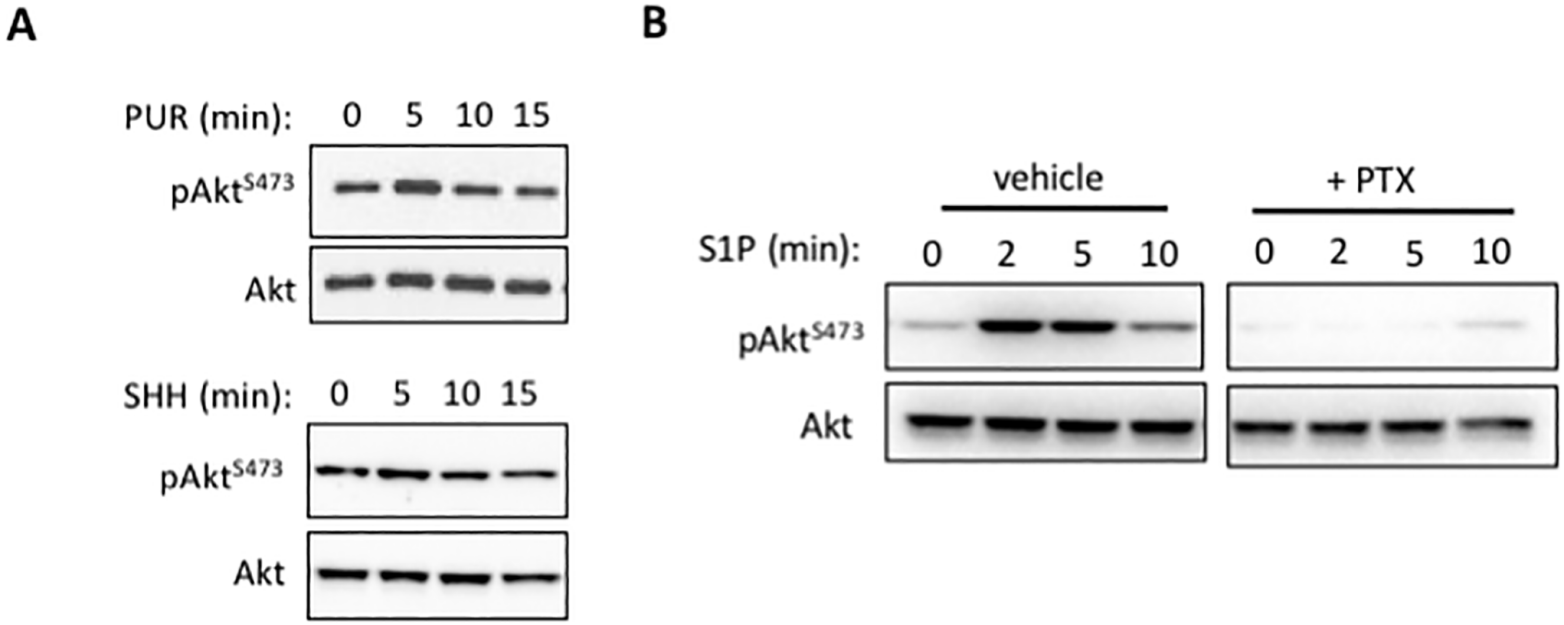
Divergence of AKT signaling profiles between Smoothened and Sphingosine-1-Phosphate Receptors. **(A)** WT MEFs were serum-starved for 24 h and then stimulated with 5 μM PUR or 2.5 μg/ml SHH for 0–15 min. AKT phosphorylation at Ser473 was determined by western blot. (**B)** MEFs were pretreated with or without 200 ng/ml PTX for 24 h before stimulation with 1 μM S1P at the indicated time points. Representative Western blots of p-Ser473 AKT and total AKT from 3 independent experiments.

We have previously reported that the pan-PI3K inhibitor LY294002 inhibits activation of RHOA and cell migration in response to SHH [13]. We reasoned that during this process AKT would be activated as a downstream target of PI3K. To our surprise, stimulation of WT MEFs with PUR or SHH did not significantly increase AKT phosphorylation (Fig 4A). As opposed to the HH pathway agonists, S1P induced a rapid AKT phosphorylation (Fig 4B) in both WT and *Kif3a*^-/-^ MEFs (S3 Fig). Pre-treatment with PTX abolished AKT phosphorylation in response to S1P (S3 Fig), indicating that it is a G_i_-dependent response.

## Discussion

In this study, we provide formal evidence that the non-canonical SMO/G_i_/RHOA pathway functions outside primary cilia. Previously, we and others had shown that a ciliary localization-deficient activated SMO mutant (SMOM2-ΔCLD) could signal to small GTPases and promote fibroblast migration [13, 21]. Here, we show that *Kif3a*-deficient fibroblasts, unable to form primary cilia and to activate the canonical HH pathway, can activate RHOA robustly after stimulation of endogenous SMO with SHH or PUR, and that KIF3A re-expression rescues ciliogenesis but does not affect RHOA activation. Since the other HH isoforms, IHH and DHH, also stimulate RHOA [16], we speculate that the three HH ligands can signal in the absence of cilia. Moreover, we show that SMO stimulation with PUR reduces cAMP levels in a PTX-sensitive manner in both WT and *Kif3a*^*-/-*^ cells. This demonstrates that SMO can efficiently couple to heterotrimeric G_i_ proteins in ciliated and non-ciliated cells. A study by Yuan *et al*. confirmed that osteoblast progenitors with defective cilia caused by inactivation of IFT80 retain, and perhaps increase, RHOA activation by SMO [22]. However, these cells retain a low level of cilia (30%) and support canonical signaling at a submaximal level, which could be sufficient to support RHOA activation. The increase in RHOA activation implied by Yuan *et al*. was not evidenced in our work here, which showed that transient rescue of ciliogenesis in *Kif3a*^*-/-*^ MEFs did not reduce RHOA activation, suggesting that the extracilliary pool of SMO is not in direct competition with the pool that accumulates at the primary cilium.

Surprisingly, while PI3K is necessary for SMO/G_i_-dependent activation of RHOA [13, 16], we found that neither PUR nor SHH significantly increased AKT phosphorylation over basal levels, unlike S1P. The differential engagement of AKT signalling by SMO and S1P receptors despite their comparable potency to reduce cAMP levels suggest that either AKT is not required for RHOA activation by those G_i_-coupled receptors or that the tonic level of activation is sufficient. Future studies are necessary to better understand the differential engagement of downstream signaling by SMO and S1P receptors.

Our findings suggest that RHOA activation downstream of SMO might be unaffected in cells with abnormal cilia, as a consequence of ciliopathies or oncogenic transformation, since many cancer cells lack primary cilia [23]. While the importance of non-canonical HH/Gi/RHOA signalling in cancer can only be speculative at the moment, there are evidences to suggest an important role. First, cancer cells rarely become quiescent (G0 phase), implying that they almost never assemble a primary cilium because the basal body is occupied in mitotic spindle formation. However, many types of epithelial cancers exhibit overexpression of SHH or IHH, which we speculate can only signal non-canonically. Second, it is noteworthy that RhoA is implicated in both lamellipodium-driven and bleb-driven migration in 3D environments, contributing to cancer cell motility (reviewed in [24]). Third, it was previously reported that the stimulatory G protein G_s_ acts as a tumour suppressor in basal cell carcinoma and medulloblastoma, which are caused by mutations in PTCH1 or SMO that results in constitutive SMO activation and high GLI transcriptional activity [25, 26]. Therefore, stimulation of G_i_ by non-canonical SMO signaling also potentiates canonical (GLI-dependent) tumorigenesis by reducing PKA-mediated phosphorylation and degradation of GLI2 and GLI3. While the contribution of non-canonical signaling in cancer has never been formally investigated, we propose that it may represent a novel therapeutic target for SMO inhibitors like vismodegib and sonidegib, approved for the treatment of advanced and metastatic basal cell carcinoma. The findings presented here are also of relevance to liver cirrhosis, characterized by activation of stellate cells, in part by a non-canonical SHH/RHOA axis [27].

In summary, our findings demonstrate that non-canonical Hh signaling mediated by events proximal to PTCH1 and SMO occur independently of primary cilia and, therefore, might be of relevance in cancer and ciliopathies.

## Materials and methods

### Cell culture

WT and *Kif3a*^-/-^ mouse embryonic fibroblasts (MEFs) were a generous gift from Dr. Pao-Tien Chuang (University of California, San Francisco, CA. The genotypes were confirmed by western blot of KIF3A. All MEFs were grown in high-glucose DMEM supplemented with 10% fetal bovine calf serum and penicillin-streptomycin and split at approximately 70% confluent. The cells used here were tested for mycoplasma.

### Chemicals

Recombinant SHH (SHH) was purified as previously described [28]. Purmorphamine (PUR) was purchased from EMD Millipore (#540220) and dissolved in DMSO at 10 mM. Sphingosine-1-phosphate (S1P) was purchased from Enzo Life Sciences (BML-SL140-001) and dissolved in fatty acid-free bovine serum albumin (BSA) at 4 mg/ml. Forskolin (FSK, Cat. #F6886), 3-isobutyl-1-methylxanthine (IBMX, Cat. #I5879), and Pertussis Toxin from *Bordetella pertussis* (PTX, Cat. #P280) were purchased from Sigma-Aldrich.

### Adenoviral vectors

pAdEasy-1 without insert (Stratagene) was used to generate the control AdV. The full-length open reading frame of human KIF3A cDNA in pCMV3-N-Myc (Sino Biological Inc.) was subcloned into pShuttle-IRES-hrGFP-1 and inserted into the pAdEasy-1 vector by homologous recombination in BJ5183-AD-1 cells. Viral particles were generated in AD293 cells following the manufacturers instructions. Titration of infective units was performed with the AdEasy Viral Titer Kit (Stratagene).

### Antibodies and western blotting

Phospho-AKT^Ser473^ (Cat#4060), phospho-AKT^Thr308^ (Cat#244F9), AKT (Cat#9272), p38 (Cat#9212), GLI1 (Cat#2643), and RHOA (Cat#2117) antibodies were purchased from Cell Signaling Technology Inc. (Danvers, MA) and used at 1:1000 dilution. KIF3A (Cat #611508) antibody was from BD Biosciences (San Jose, CA) and used at 1:1000 dilution. The β-actin antibody (Cat#A1978 clone AC-15) was purchased from Sigma-Aldrich and used at 1:10000–1:40000 dilution.

Protein samples were separated in 8% SDS-PAGE gels (for GLI1) or 10–12.5% SDS-PAGE gels (for AKT, RHOA, and loading controls) and transferred onto PVDF membranes at 250 mV for 2 h in 20% methanol, blocked according to the antibodies datasheets, incubated with the primary antibody overnight at 4 °C, washed thrice in TBST (Tris-buffered saline, 0.1% Tween-20), incubated with secondary antibodies conjugated to HRP at 1:2000 dilution for 1h at RT, washed extensively in TBST, and developed by chemiluminescence. Images of undersaturated exposure times were quantified using the NIH software ImageJ.

### Cell lysate preparation

Cell lysates for detection of phospho-proteins were prepared by scraping PBS-rinsed cells in a buffer containing 50 mM Tris-HCl pH 7.5, 150 NaCl, 1% nonidet P-40, 1 mM EDTA, 1X phosphatase inhibitor cocktail A (Santa Cruz Technology Dallas, TX), 0.5 μg/ml leupeptin, and 1X phosphate inhibitor cocktail (Catalog #PIC02, Cytoskeleton Inc., Denver, CO). Cell lysates for detection of GLI1 were prepared in 50 mM Tris-HCl pH 7.4, 300 mM NaCl, 2% Tergitol, 0.25% w/v deoxycholate, 10 mM N-ethylmalemide, 1 mM DTT, and 1X phosphate inhibitor cocktail.

### Immunofluorescence

MEFs were plated on Nunc Lab-Tek II Glass Chamber Slides (ThermoFisher Scientific), serum-starved for 24h in serum-free media, and fixed in 4% paraformaldehyde. Cells were permeabilized in 0.2% Triton X-100 and blocked in 1% bovine serum albumin. Primary cilia were stained with anti-acetylated α-tubulin (Cat #5335, Cell Signaling Technology Inc., Danvers, MA) at 1:500 dilution followed by anti-mouse-Alexa 488 at 1:300 dilution before imaging. Nuclei were stained with 2 μg/ml DAPI solution before imaging.

### RHOA pulldown assays

MEFs were plated on 55 cm^2^ dishes, serum-starved for 24 h in serum-free media, and treated with either 5 μM purmorphamine or 2.5 μg/ml Shh for 0–15 min before being lysed in 25 mM HEPES pH 7.5, 150 mM NaCl, 1% Igepal CA-630, 10 mM MgCl_2_, 1 mM EDTA pH 8.0, and 10% v/v glycerol. The pull-down assay protocol was adapted from EMD Millipore, using GST-tagged Rho binding domain of rhotekin (GST-RBD) as bait. GST-RBD was purified following a published protocol [29], with the following modifications: (1) *E*. *coli* strain DH5α; (2) 30 °C induction temperature for 2 h; (3) 10 μg GST-RBD per 250 μg cleared cell lysate. The content of RHOA in pulldowns and in the whole lysate were determined by western blot.

### Adenoviral infections

*Kif3a*^-/-^ MEFs were plated at 5.3 x 10^6^ cells per 55 cm^2^ plate (~ 60% confluency). Four hours later, cells were transduced with either AdV-control or AdV-KIF3A at MOI of 10 or 80. After 24 h, AdV-infected cells were plated in the appropriate format for GLI1 induction or RHOA activation assays and serum-starved for 48h and 24h, respectively.

### cAMP measurements

MEFs were plated on a 96-well plate, serum-starved for 24 h in serum-free media, and treated at sub-confluency with 20 μM FSK or a combination of FSK and 5 μM PUR or 1 μM S1P in the presence of 0.1 mM IBMX for 10 min before being processed for intracellular cAMP measurements using the non-acetylation cAMP enzyme immune assay (EIA) protocol with novel lysis reagents in the Amersham cAMP Biotrak EIA System (Cat #RPN2251, GE Healthcare Life Sciences, Marlborough, MA). During serum starvation, serum-free media contained either PTX vehicle or 200 ng/ml PTX. Values were normalized as a fraction of FSK (1 fold).

## Supporting information

S1 Fig Primary cilia are dispensable for RHOA activation by Sphingosine-1-Phosphate receptors. **A**. Representative RHOA pulldown assays in WT and *Kif3a*^-/-^ MEFs serum-starved for 24 h and stimulated with 1 μM S1P for 2 min. **B**. Densitometric quantification of RHOA-GTP/actin increase in response to S1P in both genotypes. *p < 0.*05* vs. t = 0; n = 3.
(TIFF)

S2 Fig Sphingosine-1-Phosphate reduces cAMP levels. WT MEFs were serum-starved at sub-confluency for 24 h in the presence or absence of 200 ng/ml PTX before treatment with 20 μM forskolin (FSK) or a combination of FSK and 1 μM S1P for 10 min. Lysates were subjected to cAMP EIA as described by the manufacturer. Bars represent % maximal cAMP production. *p < 0.*05*; n = 3.
(TIFF)

S3 Fig Akt phosphorylation in response to S1P is Gi-dependent. WT and *Kif3a*^-/-^ MEFs were serum-starved for 24 h in the presence or absence of 200 ng/ml PTX or vehicle and then stimulated with 1 μM S1P for 0–10 min. AKT phosphorylation at Ser473 was determined by western blot.
(TIFF)
