# Supplementary figures and images for: Activation of the Gi protein-RHOA axis by non-canonical Hedgehog signaling is independent of primary cilia
Source: PLoS One. 2018 Aug 27;13(8):e0203170. doi: 10.1371/journal.pone.0203170 (PMC6110505; doi:10.1371/journal.pone.0203170)

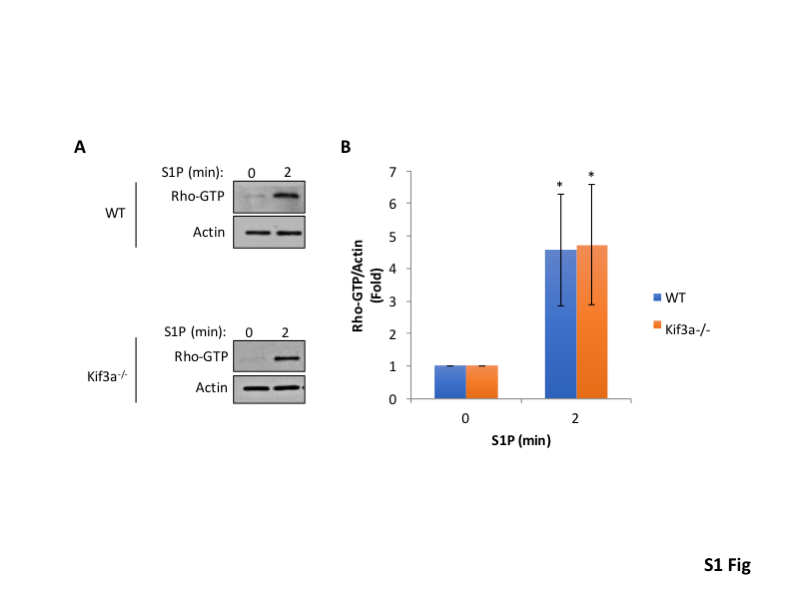

Supplement: S1 Fig — A. Representative RHOA pulldown assays in WT and Kif3a-/- MEFs serum-starved for 24 h and stimulated with 1 μM S1P for 2 min. B. Densitometric quantification of RHOA-GTP/actin increase in response to S1P in both genotypes. *p < 0.05 vs. t = 0; n = 3. (TIFF) [file pone.0203170.s001.tiff]

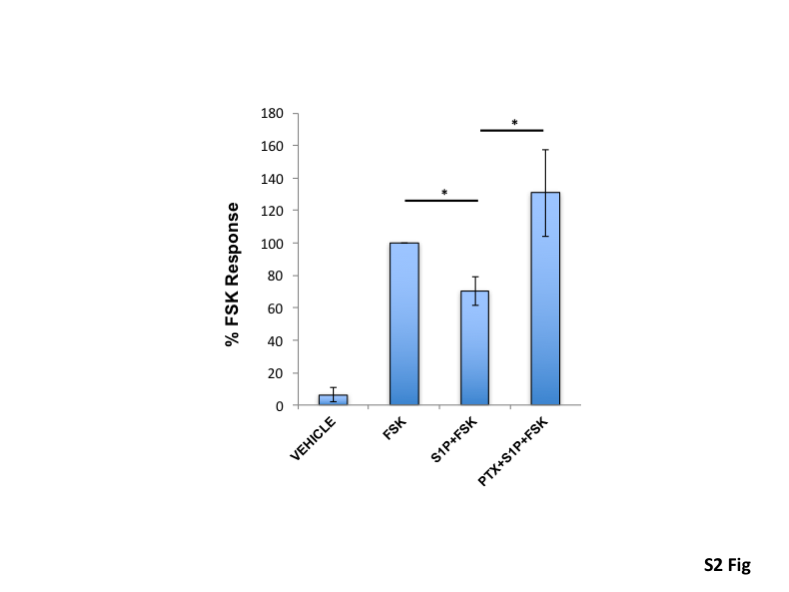

Supplement: S2 Fig — WT MEFs were serum-starved at sub-confluency for 24 h in the presence or absence of 200 ng/ml PTX before treatment with 20 μM forskolin (FSK) or a combination of FSK and 1 μM S1P for 10 min. Lysates were subjected to cAMP EIA as described by the manufacturer. Bars represent % maximal cAMP production. *p < 0.05; n = 3. (TIFF) [file pone.0203170.s002.tiff]

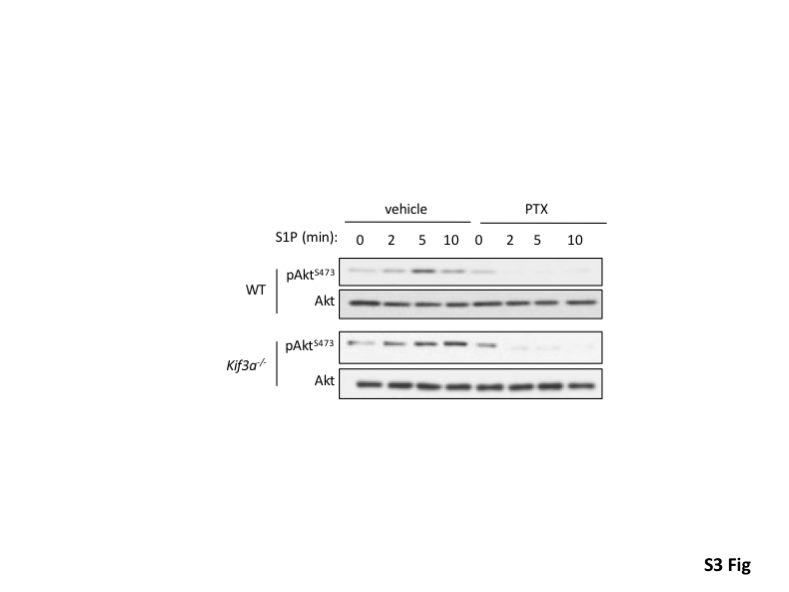

Supplement: S3 Fig — WT and Kif3a-/- MEFs were serum-starved for 24 h in the presence or absence of 200 ng/ml PTX or vehicle and then stimulated with 1 μM S1P for 0–10 min. AKT phosphorylation at Ser473 was determined by western blot. (TIFF) [file pone.0203170.s003.tiff]
